# Chemotherapy Drugs Based on Solid Lipid Nanoparticles for Breast Cancer Treatment

**DOI:** 10.3390/medicina56120694

**Published:** 2020-12-13

**Authors:** Atefeh Ashtari, Firoozeh Niazvand, Layasadat Khorsandi

**Affiliations:** 1 Department of Anatomical Sciences, Faculty of Medicine, Ahvaz Jundishapur University of Medical Sciences, Ahvaz 61335-189, Iran; dr_atefeh_ashtari@yahoo.com; 2 School of Medicine, Abadan Faculty of Medical Sciences, Abadan 61335-189, Iran; niazvandf@gmail.com; 3 Cellular and Molecular Research Center, Ahvaz Jundishapur University of Medical Sciences, Ahvaz 61335-189, Iran

Cancer is a group of diseases that include uncontrolled cell division and cell migration, as well as resistance to cell death. Breast cancer has been one of the most common cancers since the last century. It is the second leading cause of cancer death in women worldwide. However, little progress has been made in the prevention of breast cancer. The most common method of cancer treatment is the use of conventional chemotherapy drugs. However, the use of these drugs is accompanied by several limitations such as low drug solubility, high toxicity, low specificity, and low therapeutic efficacy. Another limitation of chemotherapy is the resistance of cancer cells to drug treatments.

In recent decades, nanotechnology has been developed to establish new cancer treatment strategies.

Recently, solid lipid nanoparticles (SLNs) have received a lot of attention as drug carrier systems for cancer therapy. Significant advantages of SLNs include low toxicity, high bioavailability, ability to bind to hydrophilic and lipophilic drugs, and high-scale production. SLNs can break down physiological barriers and overcome the drug resistance mechanisms of the cancer cells.

SLNs improve drug delivery to target cells using various mechanisms and controlled release kinetics. SLNs have the potential to incorporate various chemical or natural drugs. It has been shown that SLN formulations can effectively suppress different tumor growth, such as breast cancers. Diverse studies have revealed that the bioavailability and intracellular delivery of drug-loaded SLNs can be considerably increased. Hence, the therapeutic doses of chemical drugs and their side effects may be reduced by the SLN formulation [1].

There are several reports of chemotherapy drugs (hydrophilic or lipophilic) loaded into SLNs to treat breast tumors. Paclitaxel (PTX) is an effective chemotherapy drug that is at the forefront of treatment for various types of tumors such as ovarian, breast, and lung cancers. The use of this drug is associated with side effects such as nephrotoxicity and neurotoxicity. By examining the effects of the SLN formulation of this drug, reduction in renal toxicity, long-term antitumor effects, increased drug absorption, and decreased breast tumor size were observed in mice [2].

Methotrexate, as an antimetabolite drug, is effective for the treatment of breast, leukemia, lymphoma, lungs, osteosarcoma, and bladder cancers. In an in vivo study, intravenous administration of methotrexate-loaded SLNs led to the accumulation of the drug within breast cancer tissue compared with the free drug. Additionally, a considerable enhance in the life span of the treated mice with methotrexate-loaded SLNs was observed [3].

Doxorubicin, a natural factor, is commonly used as an anti-cancer compound. In addition to causing resistance in the cancer cells, its administration induces cardiac toxicity. It has been reported that doxorubicin-loaded SLNs induce high cytotoxicity and a high absorption capacity in doxorubicin-resistant human breast cancer cells [4].

Hormonal compounds such as tamoxifen have been loaded into SLNs for drug-resistant breast cancer cells. The cytotoxic and aggressive activity of tamoxifen-SLN was higher than the free-tamoxifen in the drug-resistant breast cancer cells [5].

It has been revealed that the bioavailability and intracellular delivery of other chemotherapy drugs such as capecitabine, docetaxel, epirubicin, and emodin, considerably increases with the SLN formulation. However, the efficacy of SLN formulations of these drugs has not been studied on breast malignancies. Additionally, most studies on the efficacy of drug-loaded SLNs have been performed in vitro, and in vitro studies received less attention.

Finally, considering the benefits, properties, and the high efficiency of SLNs in increasing the therapeutic effects and reducing the side effects of chemotherapy drugs, it is suggested that other anti-breast cancer drugs such as epirubicin, emodin, docetaxel, and capecitabine should be formulated in SLNs and their efficacy should be studied in vivo.

## References

[1] Bayón-Cordero L., Alkorta I., Arana L. (2019). Application of solid lipid nanoparticles to improve the efficiency of anticancer drugs. Nanomaterials.

[2] Zhuang Y., Xu B., Huang F., Wu J., Chen S. (2012). Solid lipid nanoparticles of anticancer drugs against MCF-7 cell line and a murine breast cancer model. Pharmazie.

[3] Garg N.K., Singh B., Jain A., Nirbhavane P., Sharma R., Tyagi R.K., Kushwah V., Jain S., Katare O.P. (2016). Fucose decorated solid-lipid nanocarriers mediate efficient delivery of methotrexate in breast cancer therapeutics. Colloids Surf. B Biointerfaces.

[4] Oliveira M.S., Aryasomayajula B., Pattni B., Mussi S.V., Ferreira L.A.M., Torchilin V.P. (2016). Solid lipid nanoparticles co-loaded with doxorubicin and α-tocopherol succinate are effective against drug-resistant cancer cells in monolayer and 3-D spheroid cancer cell models. Int. J. Pharm..

[5] Guney G., Cecener G., Dikmen G., Egeli U., Tunca B. (2018). Solid lipid nanoparticles: Reversal of tamoxifen resistance in breast cancer. Eur. J. Pharm. Sci..

